# Reclassification of *Catabacter hongkongensis* as *Christensenella hongkongensis* comb. nov. based on whole genome analysis

**DOI:** 10.1099/ijsem.0.004774

**Published:** 2021-04-21

**Authors:** Xiaoying Liu, Jessica L. Sutter, Jacobo de la Cuesta-Zuluaga, Jillian L. Waters, Nicholas D. Youngblut, Ruth E. Ley

**Affiliations:** ^1^​ Department of Microbiome Science, Max Planck Institute for Developmental Biology, Max-Planck-Ring 5, 72076 Tübingen, Germany

**Keywords:** whole genome phylogeny, reclassification, *Christensenella*, *Catabacter*

## Abstract

The genera *Catabacter* (family ‘*Catabacteraceae*’) and *Christensenella* (family *Christensenellaceae *) are close relatives within the phylum *Firmicutes *. Members of these genera are strictly anaerobic, non-spore-forming and short straight rods with diverse phenotypes. Phylogenetic analysis of 16S rRNA genes suggest that *Catabacter* splits *Christensenella* into a polyphyletic clade. In an effort to ensure that family/genus names represent monophyletic clades, we performed a whole-genome based analysis of the genomes available for the cultured representatives of these genera: four species of *Christensenella* and two strains of *Catabacter hongkongensis *. A concatenated alignment of 135 shared protein sequences of single-copy core genes present in the included strains indicates that *C. hongkongensis* is indeed nested within the *Christensenella* clade. Based on their evolutionary relationship, we propose the transfer of *Catabacter hongkongensis* to the genus *Christensenella* as *Christensenella hongkongensis* comb. nov.

## Introduction

*Catabacter hongkongensis* was first isolated in 2007 from the blood cultures of four patients in Hong Kong and Canada. Based on the phylogenetic positioning of 16S rRNA gene sequences and phenotypic characteristics, it was proposed as a new genus and new family, ‘*Catabacteraceae*’ [1]. The genus *Catabacter* comprises just one species, with the type strain *Catabacter hongkongensis* HKU16^T^. Based on 16S rRNA gene sequencing surveys, *C. hongkongensis* has been detected in the blood of patients with diseases such as intestinal obstruction, gastrointestinal malignancy, acute cholecystitis and hypertension in Europe, North America and Asia [1–5]. Although *Catabacter hongkongensis* was first identified in 2007, the name *Catabacter hongkongensis* was validly published in 2014 [6].

In 2012, Morotomi and colleagues isolated a novel bacterium from the stool of a healthy male adult. Based on 16S rRNA gene sequence analysis and physiological data, they named it *Christensenella minuta* DSM 22607^T^ within the novel family *Christensenellaceae* [7]. In addition to *Christensenella minuta* DSM 22607^T^, three other species have been proposed, based on additional isolates from human faeces: ‘*Christensenella massiliensis*’ Marseille-P2438 [8], ‘*Christensenella timonensis*’ Marseille-P2437 [9] and ‘* Christensenella intestinihominis *’ AF73-05CM02^PP^ [10]. ‘* Christensenella intestinihominis *’ AF73-05CM02^PP^ is proposed in a pending patent.

16S rRNA gene sequence identity (%ID) has been used to delineate genus (95 %ID) and species (98.7 %ID) cutoffs [11, 12]. The 16S rRNA gene sequence of *C. hongkongensis* HKU16^T^ has 96–97 %ID with the 16S rRNA genes of the four species of *Christensenella *, which places them in the range of sharing a genus using that criterion. In addition to sequence similarity, the 16S rRNA gene-based phylogenetic relationships of these taxa indicate they form a monophyletic clade [13].

Whole genome-based analysis with concatenated protein sequences has recently been proposed as a basis for determining the phylogenetic relationships of members of the Bacteria and Archaea [14]. Based on whole genome comparisons, *Catabacter* and *Christensenella* were annotated as belonging to the family *Christensenellaceae* in the order *Christensenellales* in the Genome Taxonomy Database (GTDB; R05-RS95 17 July 2020) [15]. Twenty-one genomes within the family *Christensenellaceae* are included in the GTDB R05-RS95 as of 1 August 2020. These include metagenome-assembled genomes and genomes derived from isolates. A formal reclassification of *Catabacter* as *Christensenella* would clarify the nomenclature of this taxon.

Here, we used comparative genomics as a basis for proposing the transfer of *Catabacter hongkongensis* to the genus of *Christensenella *. Genome sequences of six cultured isolates belonging to the families ‘*Catabacteraceae*’ and *Christensenellaceae* and four species from sister clades in the GTDB were selected for phylogenomic analysis. The average nucleotide identity (ANI) of the six genomes was compared, and a phylogeny based on 16S rRNA gene sequences was reconstructed. Based on the resulting phylogeny, we recommend that *Catabacter hongkongensis* be renamed *Christensenella hongkongensis* comb. nov.

## Methods

### Phylogeny based on whole genomes and 16S rRNA gene sequences

We based this analysis on whole genome sequences of six cultured isolates: *Catabacter hongkongensis* strains HKU16^T^ and ABBA15k, *Christensenella minuta* DSM 22607^T^, ‘*Christensenella massiliensis*’ Marseille-P2438, ‘*Christensenella timonensis*’ Marseille-P2437 and ‘* Christensenella intestinihominis *’ AF73-05CM02^PP^. General information about the genomes in this study is listed in Table 1. For the outgroup, we selected the the following species: *Clostridium novyi* NT (GenBank accession number: GCA_000014125.1), *Clostridium butyricum* DSM 10702^T^ (GCA_000409755.1), *Clostridium thermobutyricum* DSM4928^T^ (GCA_002050515.1) and *Eubacterium limosum* ATCC 8486^T^ (GCA_000807675.2). Whole genome sequences were obtained from NCBI.

**Table 1.** Phenotypic characteristics of the strains of *Catabacter* and *Christensenella* based on literature review Data for the strains are from references [1, 7–10, 31]. +, Positive; −, negative; nd, not determined. The G+C contents and N50, contig numbers, genome size and genome coverages were retrieved from the GTDB records of the strains

Characteristics	* Christensenella minuta * DSM 22607^T^	‘* Christensenella intestinihominis *’ AF73-05CM02^PP^	‘*Christensenella massiliensis*’ Marseille-P2438	‘*Christensenella timonensis*’ Marseille-P2437	* Catabacter hongkongensis *	
					HKU16^T^	ABBA15k
Gram stain	−/+	+	−	−	+	+
Motility	−	−	−	−	+	−
Catalase activity	−	−	−	−	+	nd
Metabolite utilization	Arabinose, glucose, mannose, rhamnose, salicin, xylose	Arabinose, Glucose, mannose, rhamnose, xylose, mannitol, maltose, sulphate, pine syrup, raffinose, sorbitol	nd	nd	Arabinose, glucose, mannose xylose	nd
G+C content (mol%)	51.48	52.07	50.38	51.71	48.53	48.79
Contig number	45	36	1	2	134	113
Protein count	2776	2791	2437	2430	3071	2625
Completeness (contamination) (%)	98.39 (0.81)	99.19 (0.81)	98.79 (0.81)	97.98 (0.81)	97.55 (2.97)	97.9 (3.5)
Genome size (bp)	2 940 227	3 026 655	2 560 186	2 650 850	3 203 641	2 797 114
GenBank assembly accession	GCA_001678855.1	GCA_001678845.1	GCA_900155415.1	GCA_900087015.1	GCA_000981035.1	GCA_001507385.1

We used Anvi’o version 5.2.0 for reconstructing the whole-genome phylogenomic tree [16]. Briefly, contig databases were created from the genome fasta files. Prodigal version 2.6.3 with default settings [17] was used to identify open reading frames in contigs. Hidden Markov model (HMM) profiles were used to extract the set of single-copy marker genes defined by Campbell *et al*. [18] . The best HMM hit was selected if a gene was found with multiple copies in a genome. We limited the set of single-copy core genes shared to those present in all analysed genomes and aligned the concatenated protein sequences using muscle [19]. FastTree 2 [20] was used for reconstructing an approximately maximum-likelihood phylogenomic tree with the Jones–Taylor–Thornton model [21]. SH-like local support values [22] are shown on the nodes. 16S rRNA gene sequences were retrieved from NCBI and aligned using mafft. The tree was reconstructed using the maximum-likelihood method by RAxML [23] with a general time reversible model of evolution. The phylogenetic tree was visualized using the online tool iTOL [24].

### Average nucleotide identity and phenotype predictions

We used FastANI with default settings [25] to generate a pairwise ANI comparison of the six *Christensenella* and *Catabacter* genomes. A heatmap of ANI values was generated and visualized in R [26] with the package ggplot2 [27]. Traitar [28] trait analyzer was used for phenotypic trait prediction based on genome sequences. ABRicate version 1.0.1 (https://github.com/tseemann/ABRicate) was used for the detection of genes involved in antimicrobial resistance (AMR), and the annotation was derived from the default NCBI database AMRFinderPlus.

## Results and discussion

**Fig. 1.**
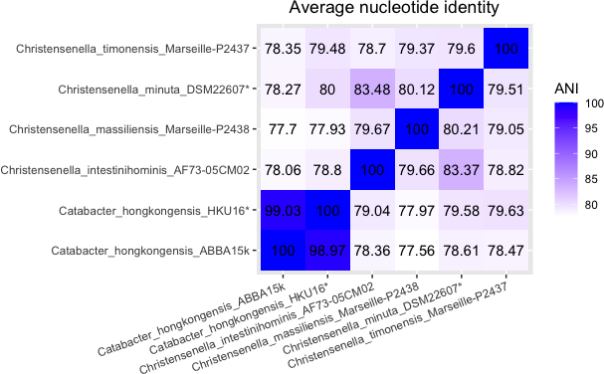
Heatmap of ANI values amongst the genomes of *Catabacter hongkongensis* strains and *Christensenella* species in this study. Type strains are marked with an asterisk.

The genome sizes of the six *Catabacter* and *Christensenella* species/strains range from 2.5 Mbp to 3.3 Mbp and the G+C content of genomic DNA from 48.53 to 52.07 mol%. Based on the pairwise comparison of the six genomes in the families ‘*Catabacteraceae*’ and *Christensenellaceae *, we observed that the ANI values of the two *Catabacter hongkongensis* strains (HKU16^T^ and ABBA15k) were >98.97 % (Fig. 1), confirming that the two strains belong to the same species. Moreover, the ANI values for the six genomes were between 77.56–83.48 %, which corresponds to the accepted ANI cut-off 95–96 % used to designate the same species [29, 30] and <83 % for inter-species ANI values [25]. ‘* Christensenella intestinihominis *’ AF73-05CM02^PP^ and *C. minuta* DSM 22607^T^ showed the highest ANI similarity values (83.48 %) between different species.

**Fig. 2.**
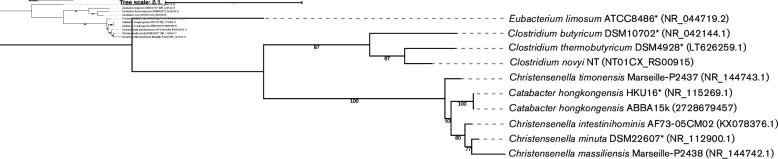
Phylogenetic tree showing the relationship of *Catabacter hongkongensis* to *Christensenella* species based on 16S rRNA gene sequence analysis. GenBank accession numbers are provided in parentheses. The gene ID number of JGI IMG Integrated Microbial Genomes and Microbiomes is provided for the16S rRNA sequence of *Catabacter honkongensis* ABBA15K. Type strains are marked with an asterisk. Bootstrap values are expressed as a percentage for 100 iterations. *Clostridium* and *Eubacterium* are used for the outgroup. The tree is rooted by *Eubacterium limosum *. Scale bar indicates 0.1 nucleotide substitutions per site.

The16S rRNA gene phylogeny shows *Catabacter* is nested within the *Christensenella* clade with 100 % bootstrap support (Fig. 2). The two strains of *Catabacter* (* C. hongkongensis* HKU16^T^ and ABBA15k) have identical 16S rRNA gene sequences. The 16S rRNA gene sequence identities between *Catabacter hongkongensis* and *Christensenella* species were between 96–97 %. Both 16S rRNA gene sequence similarity and 16S rRNA gene-based phylogenetic relationships of these taxa support that *Catabacter* and *Christensenella* belong to the same genus.

**Fig. 3.**
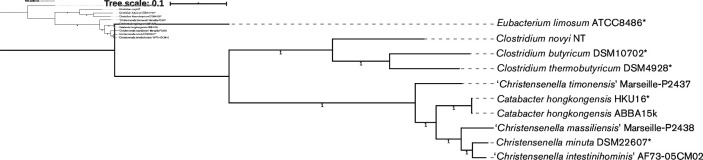
Phylogeny tree reconstructed by the approximately maximum-likelihood method showing the position of *Catabacter* relative to *Christensenella* based on 135 concatenated. core protein sequences with 51 813 aligned amino acid sites. All the nodes are strongly supported with SH-like support values of 1. Type strains are marked with asterisk. *Clostridium* and *Eubacterium* are used for the outgroup. The tree is rooted by *Eubacterium limosum* ATCC 8486. Scale bar indicates 0.1 amino acid substitutions per site.

We identified 135 protein-encoding single-copy core genes present in the genomes of *Christensenella *, *Catabacter* and the outgroup taxa. We used these 135 genes in a concatenated alignment resulting in a total of 51 813 aligned amino acid sites. In the resulting phylogenetic tree (Fig. 3), the *Catabacter* and *Christensenella* species and strains formed a monophyletic clade with high bootstrap support, indicating a shared common ancestor. The species ‘*C. timonensis*’ Marseille-P2437 is basal and forms a sister clade to the rest of the taxa in the phylogeny. The two strains of *Catabacter hongkongensis* (HKU16^T^ and ABBA15k) are, as expected based on their high ANI, on the same branch of the phylogeny. The *Catabacter* branch is a sister taxon to the remaining *Christensenella* species (* C. minuta* DSM 22607^T^, ‘*C. massiliensis*’ Marseille-P2438, ‘* C. intestinihominis *’ AF73-05CM02^PP^).

The position of *Catabacter* (and its family '*Catabacteraceae*'), nested within the *Christensenella* clade, splits the *Christensenellaceae* family and genus, such that neither are monophyletic. For the family and genus names to represent monophyletic groups, the renaming of *Catabacter hongkongensis* to *Christensenella hongkongensis* would be required. As a consequence, the genus name *Catabacter* should be reclassified as *Christensenella *.

The cultured strains of the species of *Catabacter* (* C. hongkongensis* HKU16^T^ and ABBA15k) and *Christensenella* (* C. minuta* DSM 22607^T^, ‘*C. massiliensis*’ Marseille-P2438, ‘*C. timonensis*’ Marseille-P2437 and *‘C. intestinihominis’* AF73-05CM02^PP^) have been shown to be strictly anaerobic and non-spore-forming rods with varied motility, Gram stain reaction and the catalase reaction [1, 7–10]. The different phenotypic characteristics of the species compared in this study are summarized in Table 1. *Catabacter hongkongensis* HKU16^T^ and ABBA15k strains are reported to be Gram-positive, while the four species of *Christensenella* are reported as either Gram-positive or Gram-negative. Morotomi and colleagues reported that C. *minuta* DSM 22607^T^ is Gram-negative [7], while Alonso and colleagues reported *C. minuta* stains consistently as Gram-positive [31]. Based on our Gram staining, *C. minuta* cell membranes also stained as Gram-positive, which is consistent with the observation of Alonso and colleagues. Moreover, the phenotype predictions obtained from Traitar indicate these taxa should stain Gram-positive. The Gram-variable reaction might be due to the age of the culture for staining [32].

*C. hongkongensis* strains (HKU16^T^, HKU17, CA1, CA2) and most clinical-derived isolates are reported to be motile and resistant to cefotaxime [1, 2, 5, 33] except for *C. hongkongensis* ABBA15k, which was isolated in 2016 from the blood of a patient with a fever in Sweden [34]. Strain ABBA15k showed 100 % pairwise 16S rRNA gene identity with *Catabacter hongkongensis* HKU16^T^. However, the genome of *C. hongkongensis* ABBA15k is smaller than *C. hongkongensis* HKU16^T^, and the genes coding for chemotaxin (*cheA*) and flagellar assembly (*flhA* and *MotA*) were not present in the genome of *C. hongkongensis* ABBA15k [34]. The tetracycline resistance gene *tet* was detected in the genome of *C. hongkongensis* HKU16T, but no resistance genes were detected in the genome of *C. hongkongensis* ABBA15k [34].

Screening for AMR genes of the genomes with ABRicate in this study showed that the *tet* gene was also present in the genomes of *Christensenella minuta* DSM 22607^T^, ‘*Christensenella massiliensis*’ Marseille-P2438, ‘*Christensenella timonensis*’ Marseille-P2437 and *Catabacter hongkongensis* HKU16^T^ but not in ‘* Christensenella intestinihominis *’ AF73-05CM02^PP^ and *Catabacter hongkongensis* ABBA15k. A streptomycin resistance gene (*aadE*) was also detected in the genome of ‘*Christensenella massiliensis*’ Marseille-P2438. Detailed information about AMR genes is listed in Table 2. ‘* Christensenella intestinihominis *’ AF73-05CM02^PP^ and *Catabacter hongkongensis* HKU16^T^ were predicted to be motile by Traitar. However, ‘* Christensenella intestinihominis *’ AF73-05CM02^PP^ was classified as non-motile in the original phenotypic characterization [10], which might be attributable to the growth conditions used. It is also possible that the genome of the strain may not contain all genes required for flagellar formation.

**Table 2.** Antimicrobial resistance (AMR) genes detected for the genomes of *Catabacter hongkongensis* strains and *Christensenella* species Coverage refers to the proportion of the gene in the reference gene sequence.

Strain	Contig (position strand)	Reference gene (accession)	Coverage	Identity (%)	Gene product	Resistance
‘*Christensenella timonensis*’ Marseille-P2437	FLKP01000002.1 (1477797–1479716 +)	*tet*(W) (NG_048299.1)	1-1920/1920	99.53	Tetracycline resistance ribosomal protection protein Tet(W)	Tetracycline
	FLKP01000002.1 (1480702–1481922 +)	*tet*(40) (NG_048141.1)	1-1221/1221	99.67	Tetracycline efflux MFS transporter Tet(40)	Tetracycline
‘*Christensenella massiliensis*’ Marseille-P2438	LT700187.1 (142755–144674 −)	*tet*(W) (NG_048281.1)	1-1920/1920	100	Tetracycline resistance ribosomal protection protein Tet(W)	Tetracycline
	LT700187.1 (1980989–1981855 +)	*aadE* (NG_047378.1)	1-867/867	99.77	Aminoglycoside 6-adenylyltransferase AadE	Streptomycin
* Catabacter hongkongensis* HKU16^T^	LAYJ01000061.1 (37275–39194 +)	*tet*(32) (NG_048125.1)	1-1920/1920	100	Tetracycline resistance ribosomal protection protein Tet(32)	Tetracycline
* Christensenella minuta * DSM 22607^T^	MAIR01000011.1 (54376–56295 +)	*tet*(W) (NG_048281.1)	1-1920/1920	100	Tetracycline resistance ribosomal protection protein Tet(W)	Tetracycline
* Catabacter hongkongensis* ABBA15k	No AMR genes have been detected in the genome					
‘* Christensenella intestinihominis *’ AF73-05CM02^PP^	No AMR genes have been detected in the genome					

In conclusion, both *Catabacter* and *Christensenella* include species and strains that are strictly anaerobic, non-spore forming, short straight rods and have diverse phenotypes regarding motility, Gram-staining and antibiotic resistance. The name *Christensenella* was validly published earlier than *Catabacter *. Only one species exists within the genus of *Catabacter *, while four species have been proposed for the genus *Christensenella* and the family *Christensenellaceae *. Based on our 16S rRNA gene sequences phylogeny and the genome-based phylogenomic analysis, we propose that transfer of *Catabacter hongkongensis* to the genus *Christensenella* and the species *Catabacter hongkongensis* be renamed *Christensenella hongkongensis* comb. nov.

## Description of *Christensenella hongkongensis* comb. nov.

*Christensenella hongkongensis* (hong.kong.en’sis. N.L. fem. adj. *hongkongensis* pertaining to Hong Kong, SAR, PR China).

Basonym: *Catabacter hongkongensis* Lau *et al*. 2014.

The description of *Christensenella hongkongensis* is identical to that proposed for *Catabacter hongkongensis* [1].

The type strain is HKU16^T^ (=DSM 18959^T^=JCM 17853^T^=CCUG 54229^T^).
